# On the Insanity of Dean Swift

**Published:** 1849-07-01

**Authors:** 


### THE JOURNAL
OF
### PSYCHOLOGICAL MEDICINE
AND
### MENTAL PATHOLOGY.

JULY 1, 1849.

### Analytical Reviews.

ART. I.—*The Closing Years of Dean Swift's Life, with an Appendix, containing several of his poems, hitherto unpublished, and some remarks on Stella.* By W. R. WILDE, M.R.S.A., F.R.C.S. Dublin. Hodges and Smith. 1849.

THE progress of scientific investigation clearly proves that the most extraordinary, and apparently erratic phenomena in nature, are dependent on certain fixed laws, and that no event, however remarkable it may appear, ever occurs which is accidental or irregular, or contrary to the prescribed order and harmony of the universe. If this be true, which it undoubtedly is in the material world, it is not less certain that every mental manifestation, whether of thought or feeling, which governs human conduct, and determines the character of mankind, is also dependent on psychological laws, which it is our province, in this department of science, to investigate. It has been said by Madame de Staël, that "man is complete in every individual man." Were this aphorism strictly true, the type of humanity would be so simplified, that the task of the psychologist would be comparatively an easy one; but it is far otherwise: the human mind passes through such an infinite variety of phases, and its perceptions are so modified by its organic relations with the nervous system in health and in disease, that the analysis is always attended with great difficulty. Within the whole range of biographical literature, there is no character so perplexing—none upon which a greater variety of opinion has prevailed, than that of Dean Swift. Who can account for the enormous incongruities which he exhibited in his profession, disposition, and in the conduct of his daily life? No comet, asteroid, or aerolite, ever puzzled astronomer more than his eccentricities and inconsistencies have perplexed the biographers who have attempted to describe the orbit in which he moved. But is there no clue or thread to guide us through the labyrinth in which he apparently, from some strange perversity of purpose, chose to wander? Are there no extenuating circumstances to mitigate the severe censures that have been heaped upon his memory, and which might perchance appease the shade of poor Varina, or be offered up in atonement upon the shrine of the ill-used and unhappy Stella? Or, worse perhaps than either of these cases, can no redeeming apology be suggested for his apparently indefensible conduct towards the most unfortunate of his victims—the impassioned and broken-hearted Vanessa? We answer affirmatively; for it is our belief that the wrongs which he inflicted on others as well as upon himself, "blighting his life in best of its career," are explicable upon the simple principle that he was hurried away by morbid—we should rather say, insane—impulses which he could not resist, and which eventually assumed the ordinary form of dementia, terminating in fatuity and death:—

> "From Marlborough's eyes the tears of dotage flow,
> And Swift expires a driveller and a show."

But we would fain enter our protest upon the very threshold of this inquiry against the discussion which is provoked by Mr. Wilde's book. Why, at this distant period of time, should the question of Dean Swift's insanity be mooted? *Cui bono?* It would appear as if Mr. Wilde conceived insanity to be a *stigma* upon those who unhappily are so afflicted, wherefore he sets to work with great ingenuity collating evidence, positive and negative, with a view of relieving the memory of Swift from this imaginary opprobrium. But surely insanity, or, to speak pathologically, cerebritis, whether acute or chronic, or any other morbid condition, whether organic or functional, of the brain, deranging the mind, is no more a stigma upon the unhappy sufferer than hepatitis or gastritis, pleuritis, or any other physical disease incident to humanity. Such a notion is worthy only of the superstition of the darker ages, when convulsive diseases were supposed to indicate demoniacal possession. Again: in order to appreciate fairly the evidence laid before us by Mr. Wilde, we are inevitably called upon to consider and test the rationality of Swift's conduct by reviewing certain incidents and passages in his life, better, infinitely better, consigned to oblivion.

"Save us from our friends," the living may well exclaim, who need no unwise feat of chivalry to vindicate the honour of their escutcheon; and save us, we would implore, from that species of posthumous knight-errantry which would fence with moonlight shadows round our tomb.

The retrospect of Dean Swift's biography is throughout singularly cheerless and unsatisfactory; his life altogether would appear to have been a mistake. His political career was a notorious failure; neither Whig nor Tory would trust him; he wrote, it is true, slashing libels, and his pen was feared, but he forfeited, by his tergiversation, the respect and confidence of all parties. His position in the church was equally anomalous; celebrated, like his clerk, Roger Coxe, for his facetious humour and the violence with which he indulged in party-spirit, rather than respected for the attributes which become a clergyman, it is not surprising that all his preferment ended in his obtaining the deanery of St. Patrick's, which he himself professed to consider as at best an "honourable exile." His literary success was equally anomalous; for he was known in his own day only as an anonymous pamphleteer and coarse satirist; and although it may be true that the "Tale of a Tub," "Gulliver's Travels," "The Battle of the Books," and "Martinus Scriblerus," have survived, and will always maintain their popularity from the wit and ridicule with which they abound, still all his more serious productions, which should belong to a higher order of literature—his political essays, tracts, and epistles—have long since been forgotten, and can now be regarded only as literary curiosities. "Cousin Swift," said Dryden, "you will never be a poet." Swift never forgot or pardoned this offence; but the prediction was fully verified, for Swift never rose above mediocrity in that higher description of poetry which affects the imagination and the sentiments, and has succeeded chiefly in those satirical touches which have secured for some of his isolated minor poems only an appropriate niche in the parlour of Joe Miller rather than in the Temple of the Muses.

Dean Swift was born at No. 7, Hoey's Court, Dublin, on the 30th of November, 1667, and we may remind our readers that he died on the 19th of October, 1745. After his death every minute circumstance connected with his biography, and especially with the disease under which he died, was carefully collected from the friends with whom he associated, and from Mrs. Whiteaway and the attendants who were with him throughout his long and lingering malady. We cannot, indeed, conceive clearer and more unimpeachable evidence upon any subject than we find upon the life of Swift, as recorded by

Delaney, Sheridan, Hawkesworth, Lord Orrery, Johnson, and more recently, by Sir Walter Scott. Besides which, his own journal to Stella, and his epistolary correspondence with Bolingbroke, Pope, Steele, Addison, Gay, Prior, Arbuthnot, and with many other of his illustrious contemporaries, to whom he expressed himself freely and unreservedly, have so elucidated almost every passage in his history that we have always considered that the portrait we have been accustomed to behold as that of the Dean was a true and perfect likeness. We therefore did not expect or wish to see any unskilful limner cleaning and touching up the picture, bringing shadows into relief which time and forgetfulness had mellowed down, restoring features which cannot be contemplated with satisfaction or pleasure, and resuscitating the recollection of many sad and painful events. But as we have premised, the dead, it would seem, are not permitted to rest quietly in their sepulchres. Upwards of a century has gone past, and we now find Mr. Wilde starting up to defend the memory of Dean Swift, undrawing the curtains, which, in the midst of his sufferings, concealed the aspect of his lunacy from the afflicted by-standers, and insisting, with a strange perversion of reasoning, that the Dean never exhibited any symptom of insanity.

This is not all. With more enthusiasm than judgment, Mr. Wilde describes Swift to have been a model of propriety, "the greatest genius of his age," and "one of the brightest ornaments of his country;" nay, with true Irish zeal, he calls upon "the lovers of their country to stand by him (p. 90) and defend his memory from the slights thrown upon it by the Jeffreys, Broughams, Macaulays, and De Quinceys of the day. All this sounds very chivalrous, but we are afraid that Dean Swift scarcely merited so much patriotic love, for it is well known that he disliked being considered an Irishman, and somewhat ungratefully repudiated the land of his birth. He was frequently (observes Lord Orrery) heard to say, "I am not of this vile country—I am an Englishman;" and Pope was so far deceived by his misrepresentation that, in one of his letters to Swift, he speaks of England as if it had really been the place of his nativity.* Johnson states that, "during his life the place of his birth was undetermined. He was contented to be called an Irishman by the Irish; but would occasionally call himself an Englishman."† Sir Walter Scott also remarks that when the die was cast for him to close his days in the

* Remarks on the Life and Writings of Swift, by John, Earl of Orrery. London, 1752. P. 7.

† Johnson's Life of Swift. Collected Works, by Arthur Murphy. London, 1810. P. 2.

country of his birth, his dislike to Ireland had abated nothing of its acrimony." We think, therefore, that Mr. Wilde's patriotic appeal to his warm-hearted fellow-countrymen is somewhat misplaced; but biographers, like painters, are apt to become enamoured of their subject, and hence Mr. Wilde, in amiable forgetfulness of Swift's foibles and irregularities when at college, assures us that "in early life he was of remarkably active habits, and exceedingly sober and temperate," (p. 9;) but we fear that the history of Swift's academical career by no means justifies this encomium.

In order to appreciate fairly the sanity or insanity of Dean Swift's conduct, while the malady under which he eventually succumbed was only yet in its initiatory stage, we must—however reluctantly—review some of the most prominent incidents of his life, which, in a medical and psychological point of view, are of great importance in the history of his disease.

In the year 1682, when he was fifteen years of age, Swift was admitted into Trinity College, Dublin, and we learn from Dr. Barrett, who was provost of the college, and who in 1808 published an essay on the earlier part of the life of Swift, the materials of which he collected from the Records of the University, that Swift was addicted to the vices which are too frequently incidental to a college life, at an age when young men give way unthinkingly to the indulgence of their passions, and are incapable of resisting temptation. The companions with whom Swift associated were, Dr. Barrett states, "lads of dissipated habits," and the entries in the College books show that Swift was repeatedly admonished and censured for his misconduct by the academical authorities. Between the 14th of November, 1685, and the 8th of October, 1687, he incurred, for non-attendance at chapel, neglecting lectures, and town haunting, penalties and punishments for upwards of seventy weeks. These various offences were succeeded by other delinquencies, which were considered to be so reprehensible that, on the 30th of November, 1688, the vice-provost and senior fellows issued a decree (which Dr. Barrett has extracted from the records) pronouncing Swift and three of his fellow-students guilty of exciting insubordination, using contemptuous language, and contumacy, for which offences their academical degrees were ordered to be suspended, and they were directed to crave pardon on their knees in the public hall of the college.* Sir Walter Scott seems to think that there is no absolute proof that Swift submitted to this

* An Essay on the earlier part of the Life of Swift, by the Rev. John Barrett, D.D., and Vice-Provost, College, Dublin. London : Johnson, 1808. Pp. 10, 12, 14.

despotic infliction; nevertheless, Dr. Barrett throws out no doubt upon it, but emphatically adds that "this degradation alienated Swift's affections for ever from his Alma Mater." The result of all this irregularity, negligence, and dissipation was, that when he presented himself as a candidate to be examined for the degree of Bachelor of Arts, he was found deficient, and eventually obtained the degree only by special favour, *speciali gratiâ*—"a phrase which, in that university, (says Lord Orrery,) carries with it the utmost marks of reproach. It is a kind of dishonourable degree, and this record of it, notwithstanding Dr. Swift's at present established character throughout the learned world, will always remain against him in the academical register at Dublin."* The reckless and dissipated life which Swift led at college suggested an opinion to Dr. Beddoes that the vertigo from which he suffered in after years was to be ascribed to habits of early and profligate indulgence, and he further argued that his conduct towards Stella and Vanessa, and the tone of gross indelicacy which pervades his writings indicate that degree of physical imbecility which is often consequent upon the premature exhaustion of the nervous system. Withdrawing from the university of Dublin under these discreditable circumstances, Swift determined upon obtaining the degree of Master of Arts at Oxford, and to retrieve his disgrace resolved that he would study eight hours daily. "This part of his story," observes Dr. Johnson, "deserves to be remembered; it may afford useful admonition and powerful encouragement to men whose abilities have been made for a time useless by their passions or pleasures, and who, having lost one part of life in idleness, are tempted to throw away the remainder in despair."

On the 5th of July, 1692, Swift succeeded in taking his Master's degree at Oxford. It is stated by Johnson that in the testimonials which he produced on this occasion, "the words of disgrace were omitted;" and Lord Orrery alleges that the heads of the university, by a singular misapprehension, supposed that the words *speciali gratiâ* signified a degree conferred in reward of extreme diligence and learning, instead of one attended with a dishonourable compromise; but this we can scarcely conceive, and rather believe that Swift, by unremitting industry and study, fairly qualified himself for examination.

Having obtained his academical degree, Swift entered upon the active business of life. He resided for some years with his patron and friend, Sir William Temple, went with Lord Berkeley to Ire-

* Lord Orrery, op. cit., p. 11.

land, obtained the livings of Laracor and Rathbeggan, returned to England, took a prominent part in the political skirmishes of the day, and, after propitiating the smiles of court favour, and winding his way through the sinuous paths of ministerial interest, succeeded—but not without a delay which weighed heavily upon his mind, and which he endured with the greatest bitterness of feeling—in obtaining his Church preferment. He was, in the year 1713, appointed Dean of St. Patrick's. "In point of power and revenue," says Lord Orrery, "such a deanery might be esteemed no inconsiderable promotion; but to an ambitious mind whose perpetual aim was a settlement in England, a dignity in any other kingdom must appear, as perhaps it was designed, only an honourable and profitable banishment." Before leaving Trinity College, Dublin, Swift became attached to the sister of one of his fellow-students, whose name was Jane Waring, with whom he corresponded for many years under the fictitious appellation of Varina. The young lady, it appears, acknowledged a reciprocity of affection; but prudential considerations induced her to delay giving her consent to an immediate union with her lover. Two of Swift's letters only are preserved, which afford a striking commentary upon that description of passion which, being founded upon no permanent principle of honour or affection, whiffles with the wind, and is ever ready to transfer its pretensions to the next object of attraction. "Varina's life," exclaims Swift, "is daily wasting, and though one just and honourable action would furnish health to her and unspeakable happiness to us both, yet some power that repines at human felicity, has that influence to hold her continually doting upon her cruelty, and me on the cause of it. * * * By Heaven, Varina, you are more experienced, and have less virgin innocence than I. * * * if you still refuse to be mine, you will quickly lose, for ever lose, him that has resolved to die as he has lived, all yours. Jon. Swift." This is something in the Ercles vein, for he neither died, nor married, nor broke off the connexion for four years afterwards, during which period he became acquainted with Miss Esther Johnson, whom he apostrophized in his poetry under the name of Stella. And now came the difficulty, which was, how to get rid of his Varina? His affections were estranged from her, but how were his impassioned professions of love to be cancelled? The pen of a ready writer is as easily converted into an instrument of deception, as into one conveying sentiments of disinterested generosity, purity, and love, so, with all the ingenuity of a lawyer, Swift taxed his wit to concoct a letter which should on the one hand hold out a profession of his readiness to fulfil his engagement honourably with her, and which, on the other hand, should stipulate upon conditions which he knew well she would feel obliged to reject. "Instead of either fairly avowing his inconstancy (observes Jeffrey), or honourably fulfilling engagements from which inconstancy, perhaps, could not release him, he proceeds to write to her in the most frigid, insolent, and hypocritical terms, undervaluing her fortune and person, and finding fault with her humour, and yet pretending that if she would only comply with certain conditions, he might still be persuaded to venture himself with her into the perils of matrimony. The lady, as was to be expected, broke off all correspondence after this, and so ended Swift's first matrimonial engagement, and first eternal passion. What became of the unhappy person whom he thus heartlessly abandoned after a seven years' courtship, is nowhere explained."* We have a shrewd suspicion that the breaking off of any marriage ought never to be regretted, because the fact in itself proclaims the *mésalliance* which was contemplated; but this, which is an assumption, by no means excuses or even palliates the conduct of Swift, who, while in the act of deserting his former lady-love, now invoked the Muses to assist him in winning the affections of her rival, Miss Esther Johnson.

An air of romantic interest and mystery is attached to the fate of this lady. She was the daughter of a Mr. Johnson, a London merchant, who died soon after her birth, upon which Mrs. Johnson with her two daughters, on account of her intimacy with Lady Gifford, Sir William Temple's favourite sister, became resident at Moor Park, where unfortunately Swift lived. The education of Stella had been neglected, and Swift soon took upon himself the office of St. Pierre, and began instructing his *nouvelle Heloise* in the arts of spelling, grammar, and rhetoric. He soon won the confidence of a pupil affectionately disposed, and when they separated, or were no longer in each other's company, he maintained his influence and ascendancy over her feelings by the studied assiduity of a confidential and elaborate correspondence, which, when Swift was in the meridian of his fame, could not do otherwise than flatter the pardonable vanity of a young and generous mind anxious for its own intellectual improvement, and sensible of the pleasures and gratifications which may be derived from literary pursuits. Describing her mental accomplishments, Lord Orrery observes, "she had an elevated understanding with all the delicacy and softness of her own sex. Her voice, however sweet in itself, was rendered more harmonious by what she said. Her wit

* Edinburgh Review, vol. xxvii., 1816, pp. 39, 40, et seq.

was poignant, without severity. Her manners were humane, polite, easy, and unreserved. Wherever she came, she attracted attention. As virtue was her guide in morality, sincerity was her guide in religion. She was constant but not ostentatious in her devotions. She was remarkably prudent in her conversation. She had great skill in music, and was perfectly well skilled in all the lesser arts that employ a lady's leisure." When Swift first knew her, she was about eighteen years of age; her personal appearance is described to have been very prepossessing. "Her hair," says Sir Walter Scott, whose description is derived from the best authorities, "was of a raven black, her features were both beautiful and expressive, and her form, which was of perfect symmetry, inclined to *embonpoint.* To those outward graces were added good sense, great docility, and uncommon powers both of grave and gay conversation, and a fortune which, though small, was independent." It is not surprising, therefore, that she should have received an offer of marriage from the Reverend Dr. William Tisdale, a clergyman of talents and respectability, with whom Swift lived upon a familiar and friendly footing. The proposals of her lover were made to Swift, as the lady's guardian, by whose wishes and advice she was determined to be guided, and from the evidence which Sir Walter Scott himself gives, it appears that Swift insisted on such unreasonable terms for Stella's maintenance and provision in case of widowhood, that his rival was unable to accede to them, and necessarily withdrew.

From this period Stella appears to have considered her destiny was united to that of Swift, who, having nevertheless secretly determined never to make her his wife, now cast in his mind how he could enjoy the pleasures of her society, under the roof of his own parsonage, without violating public decorum. An expedient soon suggested itself, for as necessity is proverbially the mother of invention, so vice notoriously proves itself prolific in devising a host of plausible and ingenious contrivances to conceal its own deformities. It occurred to Swift, that the presence of a third party who should never be absent when Stella was in his company, would protect the integrity of his own clerical character, of which he was exceedingly jealous, and effectually silence the whisperings of suspicion. Accordingly a Mrs. Dingle, who is described to have been a person of limited income and ordinary understanding—and who was of middle age and creditable character, was engaged to accompany Stella to Ireland; and it is believed that Swift never had an interview with her excepting in the presence of this Mrs. Dingle, to whom he pretended to pay equal attention. In the company of these two ladies he now passed all his leisure hours—to them he unbosomed all his secrets; and under the specious name of friendship he succeeded in exciting a feeling of love in the breast of Stella, which remained pure, fervent, and unshaken to the last hour of her life. That Swift promised Stella that their Platonic intimacy would be terminated by their marriage, is admitted to be more than probable; in the meantime it is certain that the conduct of Stella was strictly virtuous, and that she reconciled herself to her inexplicable and painful position by looking forward to, and cherishing, dreams of future conjugal felicity, which were destined never to be realized. "After Swift had obtained church preferment" (observes Jeffrey, severely but truly) "it is impossible to conceive any plausible excuse for his not marrying her; she was twenty-seven, and he thirty years of age, and their conjoint income was certainly ample for the support of their establishment." But, alas! the star of Swift's evil genius had again risen in the ascendant, and, forgetful of the ties that bound him to his faithful Stella, a new attachment sprang up, in which he betrayed all the heartlessness and want of principle which had already characterized the perversity of his disposition.

Upon being recalled from Ireland in 1710 to adjust a political schism between Lord Harley and Mr. St. John, afterwards Lord Bolingbroke, Swift found the family of the Vanhomrighs,* with whom he was previously acquainted, living in Bury-street, St. James's. Their house was conveniently situated, being in the neighbourhood where he lived; and here, received as an intimate guest, he passed the intervals of his political labours, displaying all those conversational charms for which he was eminently distinguished, and which rendered him the admiration of society. Mrs. Vanhomrigh was a widow lady, who had two sons and two daughters, one of whom, Esther Vanhomrigh, soon attracted the attention of Swift. She was at this period not twenty years of age, graceful, full of vivacity, and of an enthusiastic and romantic temperament. Fond of reading, she upon all occasions showed a desire for mental cultivation, which was a fatal attraction in the eyes of Swift, who, less faithful than was St. Pierre to his beloved Heloise, readily resumed the functions of preceptor; "a dangerous character," says Sir Walter Scott, "for him who assumes it, when genius, docility, and gratitude are combined in a young and interesting pupil."† This intercourse gradually became more frequent and familiar; he directed all her studies; and if not seated by her

* The name is pronounced Vannummery.—Orrery.

† Sir Walter Scott. Life of Swift, prefixed to his Works, vol. i. p. 120.

side as her literary Mentor, he was from a distance writing to her, and throwing round her confiding spirit the snares of a studiously conducted and flattering correspondence. It would seem as if Swift had discovered the secret of moving the sympathies of the female heart through the medium of the intellect; and certainly it must be confessed, that talent so evinced in the dictation of letter-writing, is apt to throw a graceful, yet dangerous charm over the emotions which may be so excited. "Believe me," he exclaims, expressing himself in French, (May 12, 1719,) "if there be anything to be accredited in this world, I think everything you can possibly wish of me, and all your desires, will be obeyed as commands, which it would be impossible in me to violate."* Then, again, he deified her in song under the name of Vanessa, and at length succeeded in inspiring an affection, which in so generous a nature proves always fatal if it does not meet with the reciprocity upon which it relies. Swift, it will be observed, was now in the forty-second year of his age; he had seen much of the world, was a shrewd observer, and knew human nature well. He could not have been ignorant of the influence which he had acquired over the destiny of Vanessa, who, in the buoyancy of youth and happiness, little dreamed that a rival, to whom his honour was pledged by obligations, if possible more sacred than marriage itself, was in the background. At length, harassed by doubt and anxiety, giving way to the impulse of her strong feelings, she determined to rend the veil, and reveal to him the intensity of her love. Nothing could exceed the anguish and supplications of her breaking heart. It was, as Coleridge finely describes—

"The voice of woman wailing for her demon lover."

Few of Vanessa's letters have been preserved, and most of Swift's, which is a significant fact, were destroyed. "Believe me," she exclaims, in a letter of remonstrance, dated Cellbridge, 1720, "it is with the utmost grief that I now complain to you, because I know your good nature is such that you cannot see any human creature miserable without being sensibly touched; yet what can I do? I must either unload my heart, and tell you all its griefs, or sink under the inexpressible distress I now suffer by your prodigious neglect of me. 'Tis now ten long weeks since I saw you; and in all that time, I have never received but one letter from you, and a little note, with an excuse. Oh! how have you forgotten me! You

* Epistolary Correspondence, in Sir Walter Scott's edition of Swift's Works, vol. xix. p. 356. Edinburgh, 1824.

endeavour, by society, to force me from you; nor can I blame you; for with the utmost distress and confusion, I find myself the cause of uneasy reflections to you; yet I cannot comfort you, but here declare that 'tis not in the power of time to lessen the inexpressible passion I have for . . . . Put my passion under the utmost restraint, send me as distant from you as the earth will allow, yet you cannot banish these charming ideas, which will ever cling by me whilst I have the use of memory. Nor is the love I bear you only seated in my soul; for there is not a single atom in my frame that is not blended with it. Therefore don't flatter yourself that separation will ever change my sentiments, for I find myself unquiet in the midst of silence, and my heart is at once pierced with sorrow and love. For Heaven's sake, tell me what has caused this prodigious change in you, which I have found of late. If you have the least remains of pity for me left, tell me tenderly. No, don't tell it, so that it may cause my present death; and don't suffer me to live a life like a languishing death, which is the only life I can lead if you have lost any tenderness for me."* In another letter, dated Cellbridge, 1720, she declares, "Solitude is insupportable to a mind which is not at ease: I have worn out my days with sighing, and my nights with watching. . . . Oh! that I could hope to see you here, or that I could go to you. I was born with violent passions, which terminate all in one—the inexpressible passion I have for you. Consider the killing emotions which I feel from your neglect, and show some tenderness for me, or I shall lose my senses."† To these and other appeals as impassioned, Swift answered by cold raillery and unmeaning sophisms, not having the courage to avow the truth and difficulty of his position, and the impossibility of his realizing the hopes which he had so cruelly implanted in her mind. Meantime, the effect of his intimacy with Vanessa became manifest in his journal to Stella, which evinced a coldness and indifference that did not escape her observation. Although her letters are not preserved, it is certain, from the allusions made to them, that they were filled with the most urgent and affectionate remonstrances. In the meantime, upon the death of her mother, Vanessa became of age, and determined immediately upon going to Ireland, for the purpose of taking possession of a small property, which her father had left her near Cellbridge.

The death of Queen Anne, at this period, broke up the Tory

* Epistolary Correspondence, op. cit., vol. 19, pp. 365, 366.

administration, and Swift, to escape the implacability of triumphant Whiggism, again returned to Ireland, where party politics were so keenly discussed, that "the ladies," said Lord Orrery, "were as violent as the gentlemen; and even children at school quarrelled for kings instead of fighting for apples."* The arrival of Vanessa in Dublin did not fail to give Swift great uneasiness, for he had studiously concealed from her his intimacy and peculiar connexion with Stella, and found himself in a labyrinth in which to advance upon either side was obviously dangerous, and from which retreat was absolutely impossible. The hour of retribution was now fast approaching. The health of Stella was visibly declining. Her pale and haggard countenance too truly depicted the inward grief which preyed upon her heart. The preference which she felt assured had been given to her rival by one to whom she had dedicated her life, with all its silent sufferings of love; the instinctive apprehension—for there is an instinct in the female mind which anticipates, by a species of intuition, impending events—with which she dwelt upon the uncertainty of her long-promised nuptials; the cold, wayward, haughty capricious mannerism of the Dean, which lacerated the feelings he still imperiously commanded; above all, the consciousness that she was living under the semblance of a cloud which threw a shade of dishonour upon her conduct;—these, and many other minor causes of annoyance, vexation, and disappointment, disturbed her peace of mind, and augmented the progress of that fatal malady which was already engrafted upon her delicate constitution. Stella died of consumption. It is well known to all medical men that the susceptibility which co-exists with this disease renders its victims peculiarly liable to be affected by mental impressions. These, when unfavourable, hasten tubercular development, and greatly aggravate the intensity of bodily suffering. Such was the lamentable condition of poor Stella; and Swift himself, with all his morbid acrimony of temper, did not, we are assured, behold the ruin of her former beauty without being in some degree affected. "His feelings at witnessing the wreck which his conduct had occasioned, will not," says Sir Walter Scott, "bear description. She had forsaken her country and perilled even her reputation to become a sharer of his fortunes when at the lowest; and the implied ties by which he was bound to make her compensation, were as strong as the most solemn promise; if, indeed, even promises of future marriage had not been actually exchanged between

* Lord Orrery, op. cit., Letter VI., p. 61.

them. In this dilemma, Swift requested his tutor and early friend, Dr. Ashe, Bishop of Clogher, to ascertain the cause of her melancholy; and he received the answer which his conscience must have anticipated—it was her sensibility to his recent indifference, and the discredit which she felt her own character had sustained from the long subsistence of the dubious and mysterious connexion between them."*

Willing to alleviate her sufferings, Swift listened to the suggestion which she made of the only course which could convince her of his sincerity, and remove her beyond the reach of calumny; and after much unworthy equivocation, he consented to the marriage ceremony taking place, upon condition that it should remain a profound secret, and that they should live separately as before. To these hard terms Stella subscribed. They relieved her mind from all scruples of the impropriety of their connexion, and they soothed her jealousy by rendering it impossible that Swift could ever give his hand to her rival. They were married in the garden of the deanery, by the Bishop of Clogher, in the year 1716. That this concession was made rather to satisfy the conscience of Swift than to ensure the happiness of Stella, may be inferred from the fact, that after the ceremony had been performed, he redoubled his precautions of never upon any occasion seeing her alone. But during this period, how fared it with the unhappy Vanessa? The rumour of Swift's attachment to Stella had not failed to reach her ears, and having now been kept in a state of painful uncertainty for *eight* years, she determined, with all the promptitude and energy of her nature, to ascertain the truth from Stella herself. Accordingly, she wrote a letter to her, requesting to know her position with the Dean; in reply to which, Stella at once informed her of her marriage; and indignant that another female should have had the right of making such an inquiry, she transmitted Vanessa's letter to Swift, and without awaiting his reply, withdrew to the house of a friend near Dublin. In one of those paroxysms of rage to which he was subjected from much slighter causes of offence, Swift rode off to Marley Abbey, and entered the apartment of Vanessa in a manner so infuriated, that she could scarcely summon courage to ask him to sit down; upon her doing which, he flung the letter down upon the table before her, and instantly left the house, mounted his horse, and returned to Dublin. "This," says Sir Walter Scott, "was her death warrant! She sunk

Scott, op. cit., p. 209.

at once under the disappointment of the delayed yet cherished hopes which had so long sickened her heart, and beneath the unrestrained wrath of him for whose sake she indulged them."

How long she survived this last interview appears to be uncertain; but she died a very few weeks afterwards, the victim of her own generous and impassioned nature, which she had not the judgment or self-possession to control. But we must not judge too harshly of so confiding and self-devoted a being. To Swift she looked up with love, awe, and admiration; he belonged, in her sight, to a higher order of mortals than she had before met with; for the daughter of Vanhomrigh, the Dutch merchant, had seen little of the world, and had no opportunity of acquiring skill in the interpretation of human character; therefore he was to her that "bright particular star" which she loved to watch in her solitary hours, and which she fondly believed would shed a protecting light over the path of her future existence. To us, the fate of Vanessa appears to claim even more sympathy than that of her sister-victims.

One more anecdote, and we must close our references to these domestic incidents, which are of essential importance in estimating the peculiar idiosyncrasy of a mind so notoriously difficult to analyse or comprehend. It relates to the death of Stella. "A short time before she died, a scene passed between the Dean and her, an account of which I had," says Mr. Sheridan, "from my father, and which I shall relate with reluctance, as it seems to bear more hard on Swift's humanity than any other part of his conduct in life. As she found her final dissolution approach, a few days before it happened, in the presence of Dr. Sheridan, she addressed Swift in the most earnest and pathetic terms to grant her dying request—'that as the ceremony of marriage had passed between them, though for sundry considerations they had not cohabited, in that state, in order to put it out of the power of slander to be busy with her fame after death, she adjured him by their friendship to let her have the satisfaction of dying at least, though she had not lived, his acknowledged wife.' Swift made no reply, but turning on his heel, walked silently out of the room, nor ever saw her afterwards during the few days she lived. This behaviour threw her into unspeakable agonies, and for a time she sunk under the weight of so cruel a disappointment; but soon after, roused by indignation, she inveighed against his cruelty in the bitterest terms, and sending for a lawyer, made her will, bequeathing her fortune by her own name to charitable purposes. This was done in the presence of Dr. Sheridan, whom she appointed one of her executors."* This anecdote comes so directly from Dr. Sheridan himself, who was an eye-witness of the facts described, that we cannot conceive any just reason for doubting its authenticity.

We must now proceed to consider professionally, and always in connexion with the anecdotes and facts recorded in this slight biographical sketch, the real state of Swift's mind. Was insanity in his case hereditary? "Certainly not," says Mr. Wilde, who designates this a most "gratuitous non-medical opinion;" but for our own part, when we recollect that his uncle, Godwin Swift, was "seized with a lethargy," after which he "was totally deprived both of his speech and memory," and died like the Dean himself, we cannot but believe that it was hereditary; nay, Swift never alluded to the death of his uncle Godwin without evincing a feeling of melancholy, and betraying an apprehension that it would be his own fate. The insanity of an uncle (a father's brother) is quite close enough to establish the presumption of hereditary transmission in any case; and we therefore do not understand the scepticism of Mr. Wilde on this point. Again: it is well known that Swift always harboured a presentiment that he should die insane. On one occasion, when walking about a mile from Dublin with Dr. Young, the Dean stopped short. We passed on, says the author of the "Night Thoughts," "but perceiving he did not follow us, I went back and found him fixed as a statue, and earnestly gazing upwards at a noble elm, which, in its uppermost branches, was much withered and decayed. Pointing at it, he said, 'I shall be like that tree—I shall die at the top.'" So also Lord Orrery remarks, "Swift certainly foresaw his fate. His frequent attacks of giddiness, and his manifest defect of memory gave room for such apprehensions. I have often heard him lament the state of childhood and idiotism to which some of the greatest men of the nation were reduced before their death. He mentioned as examples within his own time, the Duke of Marlborough and Lord Somers, and when he cited these melancholy instances, it was always with a heavy sigh, and with gestures that showed great uneasiness, as if he felt an impulse of what was to happen to him before he died." But Mr. Wilde, who appears determined to make out a case in favour of Dean Swift's sanity, attaches no importance to many facts which are entitled to much weight. "Various anecdotes," he observes, "illustrative of his eccentric habits and singular manners have been related of Swift; but as we do not think that they in any wise affect the present question, they are here altogether omitted," (p. 9.) With all

* Scott's Life, op. cit., vol. i. p. 357.

deference to Mr. Wilde, we consider that the "eccentric habits," and the "singular manners" of men alleged to be insane do materially affect the question; and it is upon this very description of evidence that commissions in lunacy constantly found their verdict. Who can read the following anecdote, for example, without supposing that the principal actor of the scene was somewhat deranged? At a period when Swift was little known, excepting to Congreve and some few literary men he had met at Sir William Temple's, he used occasionally to go into Button's Coffee House, where the most celebrated wits of that day were accustomed to assemble. "I had a singular account," says Sheridan, "of his first appearance there from Ambrose Philips, who was one of Mr. Addison's little senate. He said that they had for several successive days observed a strange gentleman come into the coffee-house, who seemed utterly unacquainted with any of those who frequented it; and whose custom it was to lay his hat down on a table, and walk backwards and forwards at a good pace for half an hour or an hour without speaking to any mortal, or seeming in the least to attend to anything that was going forward there. He then used to take up his hat, pay his money, and walk away without opening his lips. After having observed this singular behaviour for some time, they concluded him to be out of his senses, and the name that he went by among them was that of the 'mad parson.' This made them more than usually attentive to his motions, and one evening, as Mr. Addison and the rest were observing him, they saw him cast his eye several times on a gentleman in boots who seemed to be just come out of the country, and at last advanced towards him as intending to address him. They were all eager to hear what this dumb mad parson had to say, and immediately quitted their seats to get near him. Swift went up to the country gentleman, and, in a very abrupt manner, without any previous salute, asked him, 'Pray, sir, do you remember any good weather in the world?' The country gentleman, after staring a little at the singularity of his manner, and the oddity of the question, answered, 'Yes, sir, I thank God, I remember a great deal of good weather in my time.' 'That is more,' said Swift, 'than I can say; I never remember any weather that was not too hot or too cold, too wet or too dry; but however God Almighty contrives it, at the end of the year it is all very well.' Upon saying this, he took up his hat, and, without uttering a syllable more, or taking the least notice of any one, walked out of the coffee-house, leaving all those who had been spectators of this odd scene staring after him, and still more confirmed in the opinion of his being mad." This anecdote reminds us of a story told by Sir William Ellis of a complaining patient, who, hearing some persons commenting upon the badness of the weather, peevishly interrupted them, saying, "Yes, it is a very bad day; but did you ever know the Almighty send us a good one?" This story was related by Sir William Ellis to illustrate the morbid feeling of dissatisfaction and discontent which frequently possesses an insane mind; and the short and abrupt observations of Swift in the above dialogue appear to us not less significant of insanity, than the interrogatory of the poor lunatic in Hanwell Asylum.

The best educated men—politicians, physicians, literary men, artists, critics—are apt to take up crotchets; but we cannot understand Mr. Wilde contending that "Swift was not, at any period of his life, not even in his last illness, what is usually understood as mad;" while he himself has furnished us with such copious extracts from Stella's journal, and Swift's correspondence, giving a minute and circumstantial account of the early history and progress of his malady. We find that he suffered successively from giddiness, deafness, impaired sight, tremors, muscular twitchings, and loss of power in the extremities, all which are, as every tyro in the profession knows, pathognomonic symptoms of cerebral disease; yet because Swift was "never known to talk nonsense," and was not incoherent, Mr. Wilde infers that he could not have been insane. This is altogether an erroneous inference. Incoherency is no more a constant symptom of insanity than shouting and violence. Unprofessional people will have lunatics to be always like hyenas, laughing, grinning, howling, and rabid. The picture of Hogarth is ever, it would seem, uppermost in their imagination. But the truth is very different. The mind may be diseased, yet no incoherency be present; nay, the intellectual faculties may retain even a preternatural clearness and vigour. The proportion of lunatics who exhibit incoherency and violence, or who even talk nonsense, in large asylums, is much smaller than is generally supposed; so that under the present humane system of treatment, even seclusion in padded rooms is not often resorted to.

Again: Mr. Wilde thinks the composition of an epigram by Swift is an evidence of his sanity; but this we feel assured no person will admit who has had any experience in such cases. The Dean in his lunacy had some intervals of sense, at which his guardians or physicians took him out for the air. On one of these days, when they came to the park, Swift remarked a new building which he had never seen, and asked what it was designed for? To which Dr. Kingsbury answered, "That, Mr. Dean, is the magazine for arms and powder for the security of the city." "Oh! oh!" says the dean, pulling out his pocket-book, "let me take an item of that." This is worth remarking. "My tablets,"—as Hamlet says, "my tablets,"—memory put down that—which produced these lines, said to be the last he ever wrote—

"Behold! a proof of Irish sense,
Here Irish wit is seen;
When nothing's left that's worth defence,
They build a magazine!

And hereupon Mr. Wilde, with an air of triumph, exclaims, "How far this proves the insanity of its author the reader is to judge." (*Wilde*, p. 46.) This, indeed, is very sorry evidence of sanity. If Mr. Wilde will only visit Bethlehem, St. Luke's, Hanwell, or any private asylum, he will discover on the walls, windows, pillars, and posts, poetry in abundance, and epigrams too, quite as pithy as the above—

"The lunatic, the lover, and the poet,
Are of imagination all compact."

So true is this remark of Shakspeare's, that many asylums have printed the poetry written by lunatics. It is frequently done at Hanwell. At Morningside, in Edinburgh, the Crichton Institution at Dumfries, literary journals, containing both prose and verse of great merit, are regularly printed. Nay, there is often in lunacy a morbid activity of the intellectual faculties; the imagination runs wild; the perception of wit, the sense of the ludicrous, become preternaturally acute; and in such states of excitement, the lunatic will express himself, both in speaking and writing, with extraordinary vigour and fluency.

The error of Mr. Wilde appears to consist in his not understanding that insanity is a distinct disease, having its incipient and progressive stages up to the period of its maturation, or full development, when some of the intellectual faculties may be left apparently unaffected, while others are perverted and thoroughly deranged. We find that the case of Swift was, in the first instance, attended with all the ordinary premonitory symptoms of insanity; and the account which Mrs. Whiteaway, who was personally in attendance upon him throughout his illness, gives of his mental condition, can leave no doubt in any impartial mind that such was his real state.

The most minute account of this melancholy period, founded upon the evidence given by Mrs. Whiteaway, as well as upon the testimony of Mr. Deane Swift, and others who witnessed his sad condition, is given by Dr. Delany:—"In the beginning of the year 1741, his understanding was so much impaired, and his passions so greatly increased, that he was utterly incapable of conversation. Strangers were not permitted to approach him, and his friends found it necessary to have guardians appointed of his person and estate. Early in the year 1742, his reason was wholly subverted, and his rage became absolute madness. The last person whom he knew was Mrs. Whiteaway, and the sight of her when he knew her no longer, threw him into fits of rage so violent and dreadful, that she was forced to leave him; and the only act of kindness that remained in her power was to call once or twice at the Deanery, inquire after his health, and see that proper care was taken of him. Sometimes she would steal a look at him when his back was towards her, but did not venture into his sight. He would neither eat nor drink when the servants were in the room. His meat, which was served up ready cut, he would sometimes suffer to stand an hour upon the table before he would touch it; and at last he would eat it walking; for during this miserable state of mind, it was his constant custom to walk ten hours a day. In October, 1742, after his frenzy had continued several months, his left eye swelled to the size of an egg, and the lid appeared to be so much inflamed and discoloured, that the surgeon expected it would mortify; several large boils also broke out on his arms and body. The extreme pain of this tumour kept him waking near a month; and during one week it was with difficulty that five persons could prevent him from tearing out his eyes. Just before the tumour perfectly subsided and the pain left him, he knew Mrs. Whiteaway, took her by the hand, and spoke to her with his former kindness; that day and the following, he knew his physician and surgeon, and all his family, and appeared to have so far recovered his understanding and temper, that the surgeon was not without hopes that he might once more enjoy society, and be amused with the company of his old friends. This hope, however, was but of short duration; for a few days afterwards he sank into a state of total insensibility, slept much, and could not, without great difficulty, be prevailed on to walk across the room." In this state of hopeless imbecility, he is said to have remained silent a whole year. In 1744, he spoke once or twice to his servant, after which he remained perfectly silent until the latter end of October, 1745, when he expired, in the seventy-eighth year of his age.

The curiosity of strangers, we are informed, sometimes induced them to go and see this extraordinary man in this state of living death. The father of the late Lord Kinedder was of the number. He was told that the servants privately took money for gratifying the curiosity of strangers; but he declined to have recourse to that mode of gratifying his curiosity. By means of a clergyman—Dr. Lyons probably—he saw the Dean, who was unconscious of all that passed around him—a living wreck of humanity.*

We have, in addition to all these particulars, the still more satisfactory evidence of a post-mortem examination, which disclosed the pathological appearances which are usually found in such cases. Upon the head being opened, extensive serous effusion was discovered in the brain; and it is generally supposed and affirmed, that there was also softening of the cerebral substance. The internal surface of the skull, also, which was lately disinterred in St. Patrick's Cathedral, presented indications of diseased action in the vessels and membranes of the brain. "The skull," says Dr. Houston, "was thickened, flattened, and unusually smooth in some places, whilst it was thinned and roughened in others. The marks of the vessels on the bone, moreover, exhibited a very unusual appearance; the impressions of the middle arteries of the dura mater were unnaturally large and deep; and the branches of those vessels which pass in the direction forwards were thick and short, and terminated by abruptly dividing into an unusual number of twigs; whilst those of the same trunks, which take their course backwards, were long and regular, and of graduated size, from the beginning to the end of their course. We are further informed, that 'some parts in the occipital fossæ, the supra-orbital plates, and other portions of the skull were so thin as to be transparent. The internal parts corresponding to the frontal protuberances were unequal in concavity; neither was there any depression corresponding to the great prominences on the outer surface."—(*Wilde*, p. 59.) We may here remark, and the fact is anti-phrenologically important, that the immediate effect of pressure within the skull is to attenuate the internal table without producing any corresponding elevation of the external table of the skull; hence the deep pits formed by the glandulæ pacchioni, which are sometimes considerably enlarged, and the sulci occasioned by the immediate apposition of the larger blood-vessels, are limited to the internal surface of the skull, without producing externally any indication of their size or presence. In the year 1835, it became necessary to make some alterations in the aisle of St. Patrick's Cathedral; several coffins were exposed, and among others, those of Swift and Stella, which lay side by side. The following account, which Mr. Wilde has cited from Dr. Houston, of the exhumation, will probably be read with interest:—

* Sir W. Scott, op. cit. p. 403.

"The coffin, [of the Dean,] which was of solid oak, and placed transversely beneath the pillar supporting the tablet erected to his memory, and bearing the celebrated and well-known inscription written by himself, lay about two feet and a half below the flags; it was surrounded by wet clay, and nearly filled with water. All the bones of the skeleton lay in the position into which they had fallen when deprived of the flesh which enveloped and held them together. The skull, with the calvarium by its side, lay at the top of the coffin; the bones of the neck lay next, and mixed with them were found the cartilages of the larynx, which, by age, had been converted into bone. All the rings of the trachea, which had undergone the same change, were equally in a state of preservation and order. The dorsal vertebræ and ribs occupied the middle of the coffin; the bones of the arms and hands lay as they had been placed in death, along the sides; and the pelvis and lower extremities were found towards the bottom. The teeth were nearly all gone, and their sockets were filled up with bone. Six of the middle dorsal vertebræ, and three of the lumbar, were joined together by anchylosis. Several of the ribs were united to the sternum by ossification of the intermediate cartilages. The whole were evidently the remains of a very aged man. The bones were all clean, and in a singularly perfect state of preservation. When first removed, they were nearly black, but on being dried, they assumed a brownish colour. The water in which they were immersed, was remarkably free from putrefaction, and even the wood of the coffin was perfectly sound and unbroken.

"The cranium of Stella was exhumed from the vaults of St. Patrick's Cathedral, along with that of Swift, in 1835. The coffin in which it lay was of the same material, and placed in the same relation to the pillar bearing the tablet to her memory as that of the Dean; and the bones constituting the skeleton were in equally perfect preservation, though interred ten (*seventeen*) years earlier. The outline of the skull is the most graceful we have ever seen; the teeth, which for their whiteness and regularity were in life the theme of general admiration, were, perhaps, the most perfect ever witnessed in a skull. On the whole, it is no great stretch of the imagination to clothe and decorate this skull again with its alabaster skin, on which the rose had slightly bloomed; to adorn it with its original luxuriant dark hair, its white expanded forehead, level pencilled eyebrows, and deep, dark, lustrous eyes; its high, prominent nose, its delicately-chiselled mouth, and pouting upper lip; its full rounded chin, and long but gracefully swelling neck; when we shall find it realize all that description has handed down to us of an intellectual beauty of the style of those painted by Kneller, and with an outline and form of head accurately corresponding to the pictures of Stella which still exist."—pp. 57, 116.

When this exhumation took place, which appears to have been accidental, and occasioned by the repairs required in the cathedral, the British Association was holding one of its meetings in Dublin, and the skulls of Swift and Stella, as a matter of course, became each the subject of curious phrenological discussion. On the authority of Mr. Hamilton, we are informed by Mr. Wilde that the skull of Swift presented an extremely low frontal development, while "the parts marked out as the organs of wit, causality, and comparison, were scarcely developed at all." This statement we do not exactly understand, because each phrenological organ is supposed to consist of a bundle of fibres representing an inverted cone, with its apex in the medulla, and expanding until its base reaches the surface of the brain, therefore every organ must have some development. Indeed, it is only fair to state that, from the engravings which Mr. Wilde has given of Swift's skull and bust, the organ of causality appears to us decidedly large, while that of benevolence is extremely deficient—a development which we think corresponds very much with the character above described. But after all, the success or non-success of a phrenological interpretation, will depend very much on the skill and ingenuity of the expositor, which was clearly evinced in the case of Raphael, upon a cast of whose supposed skull Spurzheim and Combe lectured for many years, showing how the organology precisely corresponded with his artistical genius, his conduct to the fair Fornarina, and even the disposition of his property in his last will and testament; after which it was discovered that they had been lecturing upon a wrong skull, for the skeleton of Raphael, with the cranium connected with it, was recently disinterred in the Pantheon at Rome, and the mistake officially admitted. There is no difficulty, therefore, in reconciling any given character with any given development; and in this case the cast from the fictitious skull illustrated Raphael's character just as well as the cast from the original skull.

To revert to the psychological views which we alluded to in the commencement of these observations, it appears to us that the insanity of Swift is clearly and conclusively proved; and it is evident that the manifestations of the mind, in a state of aberration, cannot be governed by those normal principles which otherwise regulate all human actions. The constitution of the mind, when unclouded by disease, comprehends, and is influenced by certain definite psychological laws; and when these become deranged by cerebral disease, the most incongruous manifestations of thought and feeling necessarily arise. The eccentric conduct of Swift in early life indicated, we believe, that early stage of incipient insanity which unprofessional persons are always slow to recognise; for unless a man be absolutely incoherent or raving mad, the world in general, like Mr. Wilde, will not allow that he is really insane. The line of demarcation, between extravagant eccentricity and positive insanity, it must be allowed, is sometimes difficult to determine; and yet it is in this initiatory stage of the malady that the disease is most curable. We have no hesitation in ascribing the acerbity of disposition and impetuosity of temper, frequently exhibited by Swift, to morbid impulses, which he had neither the inclination nor power to control; and this view of the case we find corroborated by an able critic (probably Southey) in the *Quarterly Review*. "The Dean," he observes, "was not certainly a man to be envied. He had in him from his birth the seeds of the insanity in which, as he himself foresaw and foretold, his life was to end."* The consciousness and apprehension of the existence of this malady is often pathognomic of its being impending, if not absolutely present. The whole question at issue is, indeed, somewhat inadvertently conceded by Mr. Wilde in the following observations:—"We only wonder that Swift did not become deranged years previously; with a mind naturally irritable, a political intriguer, peevish and excitable; his ambition disappointed, his friendships rudely severed, his long-cherished hopes blighted; outliving all his friends, alone in the world, and witnessing the ingratitude of his country; while, at the same time, he laboured under a most fearful physical disease, in the very seat of reason, the effects of which were of the most stunning character, and serving, in part, to explain that moodiness and moroseness of disposition which bodily infirmity will undoubtedly produce;—we repeat, we only wonder that his mind did not long before give way.—p. 73.

We fully concur with these remarks; and although we differ from Mr. Wilde in the conclusions which he has drawn, it is nevertheless fair to state that his book is very interesting. It presents us with many new facts which throw light upon the history of Swift, and deserves a place in every well-appointed library.

* Quarterly Review, vol. li. p. 292. 1834.

